# “They don't care about people; they only care about the money”: the effects of border enforcement, commodification and migration industries on the mobility of migrants in transit through Mexico

**DOI:** 10.3389/fsoc.2023.1113027

**Published:** 2023-12-14

**Authors:** Susanne Willers

**Affiliations:** ^1^Lateinamerika-Institut, Freie Universität Berlin, Berlin, Germany; ^2^Fakultät für Sozialwissenschaften/Faculty of Social Sciences, Technische Universität Dortmund (TU Dortmund), Dortmund, Germany; ^3^Arnold-Bergstraessser-Institut, Freiburg im Breisgau, Germany

**Keywords:** border enforcement, transit migration, mobility, bodily experiences, violence, migration industries, gender and intersectionality, Mexico

## Abstract

How does border enforcement affect the mobility of migrants and refugees in countries of transit? What impact does it have on migrants' bodily experiences of mobility and their reliance on actors of the migration industry? While the externalization of borders affects undocumented people by increasing their vulnerability to violence during transit, the impact of the migration regime on the social construction of inequalities in every-day interactions and its relationship to the capacity for mobility has not been studied in depth. This article intends to bridge this gap: based on ethnographic fieldwork I conducted between 2013 and 2019, this article analyzes the relation between immigration enforcement and the mobility strategies of migrants and refugees, particularly women. It focuses on the intertwining of border enforcement and violence and their impact on people's bodily mobility experiences in transit through Mexico along intersecting lines of inequality such as race, class, gender and nationality. First, I analyze how border enforcement contributes to internal bordering, thereby increasing the vulnerability and dependence of migrants on brokers for mobility; second, it looks at the bodily experiences of women in transit and the ways in which internal bordering shapes gendered power hierarchies among actors in the field of mobility. The analysis shows how women negotiate mobility and bodily integrity in social interactions with different actors and how they face constraints resulting from the gendered hierarchies to mobility on routes of transit. Furthermore, it demonstrates how women's bodies have become a privileged site for the construction of a 'body politic' exploitable by others, since border enforcement has contributed to weakening the possibilities of negotiating mobility and bodily integrity in transit.

## 1 Introduction

The massacre of 72 migrants in San Fernando in the northern Mexican State of Tamaulipas in 2010, presumably at the hands of the drug cartel “Los Zetas” (Turati, 2013), the murder of 49 migrants in Cadereyta in the state of Nuevo León in 2012, or the killing of Victoria Salazar, a refugee woman at the hands of police officials during a routine control in Tulum, Quintana Roo, in 2021 (Lines 2021) are only some examples of the extreme violence to which undocumented migrants and refugees are exposed to in Mexico. There have been reports by national and international human rights organizations, including governmental institutions on the violence experienced by migrants [Comisión Nacional de los Derechos Humanos México (CNDH), 2009; Amnesty International, 2010; Comisión Nacional de Derechos Humanos (CNDH), 2011; CIDH, 2013; REDODEM, 2019] and even films on this subject.^1^ However, the adverse conditions and violence faced by migrants and, particularly, by women migrants and members of the LGTBIQA+ community in transit have not been adequately addressed by state policies. While extreme forms of violence get extensive coverage in the media, everyday acts of violence on migration routes receive less attention and their impact is rarely acknowledged. In this general context, rapidly changing migration control measures since 2018 have worsened conditions for undocumented migrants, including asylum seekers, and refugees.^2^ Central American migrants still make up the largest group of migrants in transit through Mexico, even though over the last few years, people from other nationalities have joined them in increasing numbers, such as Haitian, Cuban, and Venezuelan nationals, as well as extracontinental migrants.^3^ Several changes in immigration enforcement—not least in the context of the COVID-19 pandemic— have contributed to tighten border surveillance and deterrence within Mexico, and increased organized crime control over major transit routes (Álvarez Velasco, 2011). While at first glance it might seem that migrants are caught between state control and organized crime control on transit routes, the everyday experiences of migrants and refugees paint a far more complex picture. Immigration enforcement has contributed to change power balances in the field of migration through the promotion of internal bordering practices, and violence is perpetrated by various actors. Traversing Mexico on clandestine routes is an extreme bodily experience as it is marked by physical and psychological stress, by different forms of violence and uncertainty that impact the bodies of migrants. Women, children and members of the LGTBIQA+ community (Barreras Valenzuela and Anguiano-Téllez, 2022), but also men, are confronted with the effects of violent gender regimes (Connell, 1987; Hearn et al., 2020; Walby, 2020), while the geographical space of transit is controlled by diverse groups through physical and symbolic violence (Segato, 2014). In this context, the circumstances of transit seem to become normalized or taken for granted by the actors as a social rule in the field of migration (Bourdieu, 1985).^4^ There have been studies on migrants' experiences of violence while traveling through Mexico (Girardi, 2008; Castro Soto, 2010; Vogt, 2013; Willers, 2016; Brigden, 2018); scholars have also analyzed the particular impact of violence on migrant's bodies in these territories. Girardi (2008) has analyzed how women's bodies in everyday interactions on transit routes cease to be a 'resource of oneself' and run the risk of becoming an “expropriated good” (Girardi, 2008), as it is subject to a “commodification” process (Vogt, 2013) understood as an objectification which “transforms people and their bodies (…) into objects of economic desire” (Sharp, 2000, p. 293). Still people need to move and do so under the most adverse conditions. Therefore, it is important to analyze how people experience and negotiate mobility in conditions of increased immigration enforcement and violence, as this violence not only affects individuals but also has a long-term impact on families and communities.

In this article, I draw on ethnographic fieldwork and interviews with Central American women and men to analyze embodied experiences and daily bordering practices along transit routes in Mexico. I analyze how the experiences of women and their mobility strategies are related to internal bordering, especially in relation to “mobility actors” in the field, such as other migrants, migration brokers, members of crime groups and state institution officials. By bodily experiences, I refer to how migration and bordering is experienced through the body and sensations, emotions, and feelings (Longhurst, 1995). This also entails acknowledging the body as a key heuristic concept to understand the experience of migration in its social, political, and relational dimension (Scheper-Hughes and Lock, 1987). To show the social logic of gendered violence and exclusion in this border corridor, this research draws on critical border studies and feminist geographies, as well as a ‘new feminist political economy' (Anthias, 2013). Furthermore, my analysis is predicated on a micro-sociological approach based on Bourdieu (Bourdieu, 1985, 2007, also Kim, 2018), to show how different positionalities intersect and produce hierarchies of people in terms of their possibilities to access mobility and accessing rights (Mountz et al., 2012; Anthias, 2013; Lutz, 2015). These social positions are also affected by structural and xenophobic violence, racism, and discrimination against outsiders in local communities based on intersecting categories of social inequality, race/ethnicity, class and gender, ability and age, and their meaning in local settings (Anthias, 2013). By analyzing the experiences of women migrants, my objective is to contribute to our understanding of gendered forms of bordering and how the governmentality of migration and the resulting violence in the field are interconnected. Actors in the field comprise a range of individuals who take economic advantage of the need of undocumented migrants to stay put or to move, including government officials, migration brokers, and smugglers, and other service providers. The term ‘migration industry' is often used to designate the numerous types of actors involved in mobility who facilitate or constrain migration (Nyberg S1ørensen, 2013). It also refers to the different practices and a wide range of relations between these actors and the structures of migration regimes (Nyberg S1ørensen, 2013). Furthermore, migration industries and their actors do not have a fixed identity, but must be viewed in terms of place, time, and power (Lindquist et al., 2012, p. 8). Scholars have criticized the concept of migration industries as it appears to fuel the prevailing notion of unscrupulous and greedy smugglers in public discourse. These critiques have been pointing out that it obscures the impact of deterrence measures promoted by nation-states and multilevel governance which fuel the need for migrants, including refugees, to rely on intermediaries for their mobility (Zhang et al., 2018). At the same time, the concept allows to recognize that “migration industries” are not happening outside the law and are part of the policing of mobility. Therefore, it is critical to understand how migration and border regimes shape the scope of action of actors in the field of undocumented mobility (Zhang et al., 2018) and the productive meaning of violence in current migration and border regimes. The article is structured as follows: First, I review the changes to the migration regime in Mexico over the last decade and the literature relating to the body and migration for the study of violence and mobility. The second part of the article will focus on ethnographic evidence on how women engage with different actors who facilitate mobility to negotiate safety and bodily integrity. Finally, I will discuss how border enforcement has affected the mobility of migrants in recent years.

## 2 The migration and border regime in the North American migration corridor

As a transit country, Mexico is part of one of the most important transit corridors, with more than 3,000 kilometers from the South to the North in one single country (Feldman et al., 2019; Beirens, 2022). Migration control in Mexico is also marked by the geographic specificities, with a clear north-south divide between immigration control and apprehensions (Torre Cantalapiedra and Yee-Quintero, 2018). As the territory allows easy migration control in its narrowest place, the *Isthmus of Tehuantepec*, most detentions of migrants and checkpoints are concentrated in the Mexican south. In 2019, for example, 70% of all detentions were made in three southern Mexican states: Chiapas, Tabasco, and Veracruz (SEGOB et al., 2019, p. 32). Massive human rights violations and the disappearance of numerous migrants in transit have been documented by national and international human rights organizations [Comisión Nacional de Derechos Humanos (CNDH), 2011; CIDH, 2013]. In 2012, the Movimiento Migrante Mesoamericano (Meso-American Migrant Movement) estimated that approximately 70,000 undocumented migrants disappeared on their journey through Mexico (Stinchcomb and Hershberg, 2014, p. 11). Reports by NGOs and government institutions such as the Mexican Human Rights Commission (CNDH, acronym in Spanish) have shown the prevalence of different forms of violence ranging from violent assault, torture, murder, sexual violence and rape, human trafficking, and enslavement to kidnapping and blackmailing of migrants and their families [Amnesty International, 2010; Comisión Nacional de Derechos Humanos (CNDH), 2011; CIDH, 2013; REDODEM, 2018, 2019].

In the current restrictive migration and border regime in Mexico, a variety of actors take part in the field of power related to the mobility of migrants. As a conceptual frame, a “migration regime” approach helps to understand the processes involved in negotiating border surveillance through diverse practices and actors (Pott et al., 2018). The term “regime” refers to the forms this field of power is policed by diverse actors of multi-scalar governance and nation-states (Tsianos and Karakayali, 2010; Betts, 2013). It is also used to acknowledge the increasing interdependence of different actors and the emergence of new actors, such as multinational corporations and NGOs (Tsianos and Karakayali, 2010, p. 3). Tsianos and Karakayali (2010) affirm that a regime is similar to “a virtual state for certain segments of internationally intertwined political and economic processes” (Tsianos and Karakayali, 2010, p. 3–4). In Mexico, the current migration regime is marked by immigration enforcement and securitization, which are not new phenomena and are based on an increasing process of the implementation of international and transnational agreements such as the “Merida Plan” (2008), the Southern Border Program (*Programa Frontera Sur*, 2014) (Torre Cantalapiedra and Yee-Quintero, 2018), and the Cooperation Agreement between the United States and Mexico from 2019 (Ruiz Soto, 2020). Furthermore, the US government has pushed the implementation of securitization policies in transit countries in Central America through the Central American Regional Security Initiative (Meyer and Ribando Seelke, 2015) and the Third Safe Country Agreements with Central American governments (Castro Soto, 2010; Chishti and Bolter, 2020). While some of these programs officially were aimed at combating drugs and crime, they all simultaneously contributed to the containment of migration and the militarization of migration routes.

Furthermore, securitization is accompanied by a proliferation of internal borders as a deterrence practice in territories of transit and of settlement in Mexico through a “governmentality of unease” (Bigo, 2002), similar to what has been documented in many parts of the world (Mountz et al., 2012; Biehl, 2015; Hyndman, 2019). In Mexico, the new push to enforce border controls within the Mexican territory has included the introduction of new control bodies such as the National Guard in 2019 and the involvement of civilian actors such as private bus companies in internal migration control practices, which have contributed to the intensification of human rights violations against migrants (REDODEM, 2019). At the same time, administrative rules that allowed certain mobility to some groups were replaced by new procedures that increasingly restricting the mobility of migrants and refugees within Mexico.^5^ Although state policies have clearly favored the securitization of migration and contributed to the militarization of routes, increasing deportations and “permission by omission” of human rights violations have served as core deterrence strategies (Basok, 2019; Galemba et al., 2019). Between the years 2002 and 2017, Mexico deported 1.9 million people from three Central American countries, compared to the United States deporting 1.1 million (Flores et al., 2019). This tendency has also led to a large number of people who are unable to reach the southern US border to claim asylum applying for refugee protection in Mexico. Refugee applications in Mexico have steadily increased since 2013 [Secretaría de Gobernación (SEGOB) and Comisión Mexicana de Ayuda a Refugiados (COMAR), 2017]. In 2021, Mexico had the third highest number of refugee applications in the world, with 132,700 in 2021 (UNHCR/ACNUR, 2021, p. 3). This is consistent with the trend that low-income countries in the Global South are hosting 83% of the world's refugee population, which accounts for 74.2 million forcibly displaced persons (UNHCR/ACNUR, 2021, p.2) and can be seen as a consequence of the shift in enforcement policies from the Global North to the Global South through securitization policies.

## 3 The embodied experience of migrants in border regimes

Traversing the North American migration corridor through Mexico is a bodily experience, as migrants face a difficult journey along clandestine routes which they undertake by foot or on freight trains, traversing rough territories which pose many risks and dangers without the necessary physical preparation and protection. They try to reach a safe space without the most basic secure access to food, water, a toilet, or appropriate shoes. Many mothers travel with their small children, caring all their belongings in a small backpack. As has been shown, transit is accompanied not only by the fear of suffering bodily harm and traumatizing forms of violence, but also by psychological stress and deprivation (Basok, 2019). The precarious conditions faced by undocumented migrants and refugees do not stop states and state agents from prosecuting and questioning their reasons for fleeing, nor do they prevent crime groups from preying on them. On the other hand, migrants have agency and engage in complex negotiations with actors on the ground. These processes themselves then “create opportunities to exercise agency” (Deshingkar, 2019, p. 2638).

Analyzing border policing and its effects on migrants' bodily experiences in the process of transit migration through Mexico requires mapping the actors who take part in the production of the social space. In the case of the North American migration corridor, this means acknowledging the “inconsistencies and ambiguities” (Fassin, 2011, p. 218) produced in the migration regime and by actors on the ground. Furthermore, internal bordering is produced by “everyday bordering”, which brings the border into social relations, social institutions, and local life (Yuval-Davis et al., 2018). Thus, bordering is conceptualized as practices that are located and constituted in the specificity of political negotiations, that shift and are “contested between individuals, groups and states as well as in the construction of individual subjectivities” (Yuval-Davis et al., 2018, p. 230). Mexican state and government officials contribute to enforcing migration control on the one hand, and the provision of humanitarian assistance on the other, but they are also involved in various activities related to the exploitation of benefits or the participation in crimes against migrants (Galemba et al., 2019). It is therefore imperative to analyze forms of violence exerted by different actors and how different forms of violence impact the bordering practices observed. Among them, forms of “organized violence” are understood as “the use of force in a collective, organized way (…) perpetrated by constituencies like nation states as well as collective or corporate actors, legal and illegal, with varying levels of legitimacy” (Pries, 2019, p. 35). In the context of the Mexican “war on drugs” (since 2008), this means to analyze how structural and political violence against migrants has contributed to convert undocumented mobility into one of the main incomes of groups of organized crime (Durand, 2011) and local communities on transit routes.

For those who move as undocumented and “illegalized”, the body becomes a central site of bordering. Thus, the body is a key concept to analyze the bodily experience in the context of forced migration processes. Scheper-Hughes and Lock (1987) distinguish between three different perspectives of the body as a heuristic concept for understanding social processes evolving around it in societies: (1) the phenomenological experience of the individual body understood as ‘body-self', (2) the social body in a structuralist tradition, which looks at the representational and symbolic uses of the body, and (3) the ‘body politic' in poststructuralist epistemology which refers to “the regulations, surveillance, and control of bodies” (Scheper-Hughes and Lock, 1987, p. 8). In a structuralist tradition, Bourdieu (2007) has shown the relational logic of violence. Social hierarchies are also constructed by forms of symbolic violence exerted in gender relations and which construct social differences between people based on normalized forms of difference and othering (Bourdieu, 2007). The poststructuralist approach to the study of the body tells us “how certain kinds of bodies are socially produced” through “codes and social scripts” that contribute to the “domestication of the individual body according to the needs of the social and political order” (Scheper-Hughes and Lock, 1987, p. 8, 26). To understand the subordination of the individual body in the “body politic”, it is crucial to understand how violence, torture and subordination are interlinked with the economic processes of exploitation, especially in the context of postcolonial processes of exploitation (Foucault, 1995/1977; Walters, 2015). Incidents of torture, murder, and massacres against transit migrants on transit routes show many similarities to these extreme forms of violence described by Taussig (2004) as “cultures of terror” that display violence to maintain the established (post-)colonial order and to ensure economic hegemony. At the level of the body, violence then produces ‘docile bodies' (Scheper-Hughes and Lock, 1987) and ensures cheap and exploitable labor (Mezzadra and Neilson, 2013).

In the context of Mexican transit routes, a complex system of interlinked actors participates in the economic exploitation of the need for mobility of migrants, who are mainly fleeing violence and the effects of economic deprivation in their countries of origin (Orozco and Yansura, 2014; Willers, 2016). Subordinated and racialized migrants' need for labor, housing and mobility then become a powerful driver for the local, national, and transnational economy. In the process of commodification of the bodies and existences (see Vogt, 2013), these acts of violence against migrants in transit then produce wealth as they bring the border to the bodies. Unequal power relations and exclusion are inscribed in the bodily experience of migrants and refugees in transit. This has been called a “border effect” in the lived experiences of migrants and refugees, as Idler has outlined in the case of Colombia (Idler, 2019). The “gendered border effect” intensifies the logic of borders and “the logic of violent non-state group interactions” in form of a “*double impact”* of armed conflict and “refugee and migrant crisis” on women's bodies (Zulver and Idler, 2020, p. 1123). While systematic violence against migrants and refugees in transit throughout Mexico has been well documented [Comisión Nacional de Derechos Humanos (CNDH), 2011; CIDH, 2013; REDODEM, 2019]; the rationale for (sexual) violence against women and men has not been sufficiently explained and must be understood in the broader context of patriarchy and violence against women. In this regard, Segato (2014) has shown how violence against women works as a form of “pedagogy of cruelty” by different groups and fractions who take part in new forms of war (Kaldor, 2014) and seek to establish dominance over a territory by exalting violence against gendered and feminized bodies of the opposite, or subordinated, group. Violence then becomes a tool of terror and control over a large group of people, who can then be exploited in many ways. In the context of the Mexican “war on drugs” (since 2008) and the militarization of migration routes, transnational criminal groups (Correa-Cabrera et al., 2015), control transit spaces by inflicting suffering, fear, and violence. This rationale is visible when we analyze the narrations of migrant women who have been crossing the transit routes through Mexico.

## 4 Methods

This article draws on data collected through ethnographic fieldwork for my Ph.D. thesis (2013/2014) (Willers, 2017) and postdoctoral research (2018/2019). During those years, I visited several urban centers along migration routes, including Tijuana and Mexicali, two border towns on the northern Mexican border with the United States, Tapachula in the South, and Mexico City in the center of the country. The research was based on a qualitative methodology following a grounded theory approach (Strauss and Corbin, 1996) and problem-centered interviews (Witzel, 2000) with migrants and refugees, as well as expert interviews with social workers at NGOs and institutions. Throughout those research periods, I interviewed 57 women and 6 men. Additionally, I interviewed 26 experts from various institutions who worked in fields related to the topic of migration in Mexico and had informal conversations with migrants and refugees, which were not recorded, but registered in the research diary and shorthand. The interviewees came mostly from four Central American countries: El Salvador, Guatemala, Honduras, and Nicaragua. The main goal of the inquiry was to register the gendered experiences of transit migration, social interactions in the field and survival strategies. Migrant women and families were approached in NGOs or migrant shelters that provided humanitarian aid to people in mobility. Interviewees received information and explanations about the goal and objective of the study and were asked to provide their informed consent for their interviews to be recorded and analyzed. All names have been changed to guarantee anonymity. In researching forced migration processes, I was aware of tensions, conflicts, and vulnerabilities that could arising from potential power hierarchies. I tried to counteract these tensions through open communication about my objectives, a respectful attitude toward the concerns and problems, and discretion in the content of our conversations. Also, even if I did not research directly on the brokers on mobility at that time, their presence was also evident in the narratives of most of the people interviewed and thus the existence of an economy related to undocumented migration/mobility.

## 5 The bodily experience of transit: ethnographic evidence from the routes

Although the process of securitization of migration routes across Mexico is a long-standing phenomenon (Castro Soto, 2010), increasing border enforcement has also helped integrate internal borders into transit routes, thus changing the balance of power between the actors on the ground, increasing pressure of surveillance, and limiting choices of migrants. As transit conditions change, so do the experiences of transit and coping strategies of migrants on the routes. The analysis is structured as follows: I will show, first, how people interact with different actors who enable mobility along *securitized* migration routes and how migrant women and their families try to reduce the risk of gender-based violence along migratory routes, second, I will show how migrants who have repeatedly traveled have experienced the changes on routes over time and how this has affected mobility in recent years. Although I cannot go into detail about the impact of all border security measures, I will outline some general trends in their impact that can be observed in the narratives of migrants transiting Mexico.

Central American migrants and refugees move because of complex constellations of causes related to violence in their countries of origin that range from gender-based violence and intimate partner violence to overall social violence and persecution by organized crime groups (Orozco and Yansura, 2014; Stinchcomb and Hershberg, 2014; Willers, 2016). Those who are forced to flee due to threats mostly migrate without economic capital, and not all of them can count on reliable social contacts of family and friends in transnational networks. However, even people who travel without means try to adapt their strategies to the conditions they encounter on the road, which are often characterized by experiences of violence and the fear of suffering aggressions. Traditionally, “*coyotes*” or smugglers^6^ have been hired as professional service providers who know the routes and can reduce potential harm and increase the success of the journey (Stone-Cadena and Álvarez Velasco, 2018; Zhang et al., 2018; Torre Cantalapiedra and Hernández Campos, 2021). But prices for guides, also called “*coyotes*” or “polleros”, have been rising in the last decade and vary according to the country of origin and travel distance (Gonzalez-Guevara, 2018; Gandini, 2020).^7^ Many people cannot afford to pay for the whole trip from their home to the destination. Instead, they attempt to make the journey in smaller stages, trying to use guides only for certain segments considered more difficult than others (see also Gonzalez-Guevara 2018). This has changed hiring modes and entails a higher risk of connecting with ‘false coyotes' or people who would try to cheat or lure their clients, as it is more difficult to know their reputation (Stone-Cadena and Álvarez Velasco, 2018; Torre Cantalapiedra and Hernández Campos, 2021). In addition, the emergence of large caravans of migrants in 2018 and the COVID-19 pandemic have shown that social networks are playing an increasingly important role in accessing information (Gandini, 2020; OIM, 2023). However, it is unclear to what extend this will change the way people access intermediaries for mobility. For example, whether social media can provide the same quality of contacts to networks or replace traditional forms of smuggling. Ostensibly, they open the way for a depersonalization of smuggling services, but this risks an increase in amateur services by less experienced or even “false smugglers” (Stone-Cadena and Álvarez Velasco, 2018, p. 206). Although there have been significant changes in people's access to IT devices and access to information and communication technologies over the past decade, during the period of my fieldwork, most people still relied primarily on simple cell phones, rarely smartphones, and many cases of robbery were documented, as these items represented wealth that people were quickly deprived of.

The choice of transportation depends primarily on people's economic resources and their need to remain ‘invisible'. Freight trains are the first choice for undocumented migrants without economic means who need to move and avoid checkpoints along major highways. However it is not a cheap way to move as it is known that people need to pay a fee to the groups who control the route. In 2013, I met Karen and her 3-year-old son in a shelter in Mexicali, where she waited to be “returned”—this meant deported by the Mexican migration authorities—to her home country, Guatemala. She decided to turn herself in after suffering gender-based violence by the son of a woman who gave her shelter. Previously, the smuggler who had brought her from the southern Mexican border had abandoned her at the U.S. border. He had decided to pass her brother first and had not returned to pick her up. In addition, her aunt had stopped sending money. Even though, she was sad about not having been able to cross the border; she was not willing to endure any more violence. Karen recounted her experience along the journey after she had left home with her brother and her little son and had crossed Mexico on the freight trains from the South to the North.

Yes, not all of them [migrants] come by train. Because most of them, if they have enough money to pay a good *coyote*, they don't come by train. Those of us who don't have a lot of money come by train. But the majority comes by train. You see everything on the train. You see all kinds of people and people from many countries. We met Cubans, Salvadorans, Hondurans, Nicaraguans, yes. And Guatemalans, we met quite a lot. Uh-huh. (…) I came with my brother and the one who brought us, the *coyote* [guide]. We caught him in Tecun Umán [Guatemalan border town], at the border, we caught him there (…) we paid him. He was paid there and here [at the US border] he had to be paid so that he could pass us upwards. In Guatemala he received 25 thousand quetzales… (…) So it was about 35 or 30 thousand pesos.^8^ (…) and we paid it because the child was coming with us, so we said ‘they are not going to hurt us'. But no. I think we are all exposed to that. They don't care about people, they care about money. How much is a person worth? Uh-huh. But I do think this is, it has been very difficult [sic] (Karen, Mexicali, 2013).

Having traveled more than 3,700 kilometers on the roof of a freight train, her experience was shaped by the precarious conditions of her journey. Knowing that Mexico is a difficult territory to cross and traveling with a small child, she hired a “*coyote”* right at the border between Guatemala and Mexico to be “safer”. Paying a guide not only meant escaping state control, but also potential attacks by criminal groups and gangs, reducing potential harm and assuring physical integrity. Women and children are believed to be at disproportionate risk of being targeted by criminals and becoming victims of violence, including sexual violence, rape, kidnapping, and extortion by various groups. On these routes, a plurality of actors gets on the train, including local crime groups and state actors involved in raids. Obeying the market principles of “the higher the risk, the higher the price”, smugglers then charge higher amounts for women and children, although, as the interviewee explained afterwards, this is not a guarantee for protection. By saying “we are all exposed”, she also indicated that even smugglers as facilitators were exposed. As she explained later, they must pay a fee to the groups that control transit routes and trains, or they risk being punished through violence, including sexual violence, against themselves or their clients, which then affects their reputation and their smuggling business. She expressed her disbelief at the unbearable logic of indifference toward the value of people's lives prevalent in train interactions: “They don't care about people; they care about money. How much is a person worth?”, acknowledging that even though people pay smugglers in the hopes of being safer, there is little security possible. Regarding the ability to provide protection, Karen narrated the following incident:

I: And the coyote didn't take care of you?

K: Yes, he did; the thing is that on the train, we are exposed to many things. Kidnappers get on,… So he can't do much, just say, “Don't take her. Or don't do that to her.” But that's all. With a gun, you can't move. With a gun, you keep quiet and say: “Ok.” Uh-huh. In one train, a young man got on, covered his face, and everything. You could only see his eyes, his nose and his mouth, and he asked me who I was traveling with. And the men were silent. And I thought “They're going to put me down.” (…) And he wanted me to get off. He wanted me to go to where he was. So, I told him that I had a sore foot. And he told me: “Wait here! Wait!” But since it was nighttime, he left. When he came back, I wasn't there anymore. I was hiding in that hole in the train. And the boys said: “No, there is no woman!” [sic] (Karen, Mexicali, 2013).

Not only are people mostly charged high amounts of money, as Karen, but still women risk their lives and bodily integrity in this trajectory. Her narration also showed how women have become a particular target in the logic of commodification of migration routes. But sometimes also a bail to put pressure on the group of migrants. As she explained: “*The men were scared out of their wits. They were all very scared. Because when they were going to put me down, they weren't going to let them put me down. So, as he was armed… But thank God no! (…)”* (Karen, Mexicali, 2013). The men traveling with her were also scared because they felt responsible to protect her. Thus, the border is experienced by women through their bodies and puts them at risk of becoming targets of the various actors involved in controlling transit routes. These include not only organized crime groups but also government officials, other migrants, and migration brokers who exploit the subordination of migrants and refugees through border control. These structural, political, and cultural conditions of transit in the context of increased immigration enforcement turn into a kind of “unspoken rule” (Bourdieu, 1997) on migration routes that undocumented mobility entails high risk and little protection. Migrant women need to negotiate their safety and try to adapt their strategies for transit. In the undocumented migrant community, there is solidarity and mutual help, but there is also competition, fear, and betrayal. In the social field of undocumented migration, ambivalence prevails in relationships between people, where one and the same person can potentially take on different roles, being helpful and showing solidarity for some, but taking advantage of the situation for others. This also affects the relationships between migrant men and women on the routes. In patriarchal logic, women find “help” by traveling with men who are supposed to “protect” them from harassment by other men. However, the women interviewed recounted that they would avoid traveling with men and asking for their protection because they felt obliged to “pay” the favor of supposed protection with sex'. An interviewee asked if traveling with men would provide her with protection and answered: “*No, no, no. Because today men are no longer the same. The longer time passes, the uglyer they get, the rougher they get. If they do you a favor, they want to charge you for it, and so on. They start extorting and bothering you, so they don't.”* (Maria, Tapachula, 2013). This also means that women who travel alone are trapped in gender, structural, and political violence, and in a patriarchal logic that limits their mobility and expects them to “pay” a different price for mobility and “protection”.

Regarding the changes in securitization and the balance of power between actors of the migration industry on transit routes and borders in recent years, Andrea, a 54-year-old migrant from El Salvador, shared how she experienced these changes. She traveled these routes several times from South to North with her husband. She first arrived in the United States in 1986 but was deported in 2004 and forced to go through Mexico several times without being able to cross the U.S. border again. She remembered the changes in Laredo, Texas, when “Los Zetas”, a notorious drug cartel known for its use of extreme violence, took control of the northern Mexican border.

The “Grandfather” was the boss of the “*polleros*” and they killed him, and after they killed him we left because it got ugly, because of those who were there on the river. Because when I first arrived in Laredo there was no “Los Zeta” guarding the river and collecting fees, in other words, you could pass through and there was no problem…. (…) Well, it was around 2000, and then when we went back to try to cross there by the river, by the *Rio Bravo* in Laredo, no longer, there were already a lot of “Zetas” there, you had to pay them a fee. No, I said, I better go back. By that time, they had already killed “the grandfather” [sic] (Andrea, Tijuana 2013).

As interviewees reported, government control and the increasing control of organized crime groups on transit routes often go hand in hand. Also experienced migrants who know the routes, as they have already traveled them several times, are affected by the changed conditions and the prevailing logic of violence in the transit zones. Rapidly changing border enforcement and securitization measures are also affecting the diverse mobility resources of migrant communities, as they make access to information more difficult. Reliable information is mostly provided by social networks and is an important prerequisite for safer transit; yet, with the increasing speed of changes in border enforcement, this is becoming a scarce resource. In addition, migrants and refugees have different access to confidential networks. People who can plan their migration in advance could potentially seek out and rely on more trustworthy smugglers than those who were forced to leave quickly without the ability to prepare. Smuggling services with a reliable reputation are usually contacted from countries of origin and destination. But people without many resources and strong networks rely on *coyotes* they find along migration routes, or in migrant shelters by the recommendation of other migrants. However, it poses the risk of trusting people who turn out to be scammers (*estafadores*). Eduardo, a seasoned El Salvadoran migrant interviewed in southern Mexico, traveled accompanied by his two nieces. Eduardo felt responsible for their safety. After having been told by others that they were an easy target for rape and assault, he felt afraid to travel the clandestine routes with both women. He was scared and thought that a guide would help him solve the problem. Finally, he trusted a couple who would serve as guides to cross immigration checkpoints, but the group was stopped anyway. After their detention by the Mexican Migration Institute (INM), the same couple then tried to extort money from their family in El Salvador, saying they would have to post bail to be released, while he and one nice were deported. Their experience showed how the threat of sexual violence is a powerful barrier to the mobility of women that also opens the way to further forms of extortion. These stories of betrayal are an everyday occurrence on migration routes, and people receive constant advice at migrant shelters or NGOs not to trust people who offer to help as smugglers or “coyotes”. However, it is essential to gather information from others to proceed, even if they are mostly strangers. Cities and communities with migrant shelters are therefore “spaces of possibilities and spheres of orientation” (Vigh, 2007) as important places of recreation and information sharing, where people meet or wait for their *coyotes* or *polleros*, often recommended by their families. The humanitarian infrastructure and migrant shelters are unmissable, necessary places for migrants to make their way through the ever-changing and complex conditions of transit. The exchange of information between migrants in local spaces is an important mechanism to guide the movement of people and mitigate the impact of uncertainty. This has been discussed by Parrini and Flores (2018) as a form of resistance and a strategy of “collective production of coordinates for orientation” through the construction of oral maps, which help migrants navigate their way. While smuggling services in the best cases connect places of origin and destinations, under current conditions the transit has become an incalculable risk for migrants even for those who can pay a facilitator.

In current circumstances, the struggle for survival is not just a metaphor, but a lived reality for people trying to escape extreme violence and poverty. In Mexico, undocumented migrants have to pay for everything, even things that are allegedly free, such as boarding freight trains, asking for alms in public space, or even participating in the survival economy by selling sex (Álvarez Velasco, 2011; Stinchcomb and Hershberg, 2014; see Muñoz Martínez et al., 2020).^9^ The violence perpetrated against transit migrants, especially women, is normalized in the form of symbolic violence on the level of everyday interactions and public discourses, which mostly see women as victims and underestimate their agency, as well as their need to migrate in order to survive and to maintain their families. At the same time, it renders invisible the political, economic, and cultural structures which enforce the gendered logic of women's subordination. Furthermore, public policies ignore victims' rights to safety and protection and contribute to revictimization, which is one of the reasons many women who have suffered violence do not report these acts to authorities (Willers, 2019a,b). As the analysis showed, the body is the vehicle through which the migrant journey is experienced, as well as a major tool for agency. Therefore, border enforcement has contributed to the construction of migrant bodies through a ‘body politic' (Scheper-Hughes and Lock, 1987) that turns their bodies to objects exploitable by others and by charging money for their bodily integrity. This situations leads to gendered inequalities of mobility and to an increasing need to draw on “reverse” remittances (Mazzucato, 2011) from families in home countries for migrants to access migration services provided by actors in the “migration industries” to make their way to the US. On the other side border enforcement has weakened the possibilities of migrants to negotiate their bodily integrity and the conditions of transit with actors that facilitate or control mobility by contributing to power- imbalances on routes of transit.

## 6 Discussion

In this paper, my aim was to explore the ways in which border enforcement has contributed to shaping the bodily experiences of transit in the experiences of migrant women. In particular, I was interested in how women experience internal bordering and how it shapes the power hierarchies of actors in the field of mobility. The analysis showed that border enforcement has had concrete effects on how migrants negotiate their safety and bodily integrity in the context of undocumented mobility, and that bordering is experienced through violence and terror on migration routes. The findings display three aspects relevant to the study of bodily experiences of migration in transit. First, the current transit conditions faced by undocumented migrants impact their interactions with actors in the field of mobility when negotiating the terms of mobility and safety. In view of the high dynamics of changing actors and an increasing militarization of transit routes, mobility itself and the negotiation of its conditions have become more difficult. Changing actors and new bodies of migration control, such as state agents of newly created corporations, or private security of bus and train companies, contribute to modifying power dynamics in the field. Therefore, the militarization of transit routes driven not only by the state but also by other groups of “organized violence” (Pries, 2019) has contributed to (re)shaped practices of “migration industries” and their actors and deepened the complexities of negotiating between clients and brokers. The ambivalent positioning of migrants, in hierarchical relationships toward the state, state actors, and actors of undocumented mobility during transit, has been analyzed by Coutin (2005, p. 196). She has shown how immigration enforcement contributes to positioning migrants in a “liminal political-legal space” of mobility where they are “simultaneously in and out of space” (Coutin, 2005, p. 196) and therefore extremely vulnerable to violence and exploitation. This is also the case under conditions of heightened border enforcement in Mexico (Galemba et al., 2019). Second, the ability of undocumented migrants to negotiate their transit with the help of smugglers or *coyotes*, which are hired by migrants to reduce risks, is weakened, as there is a plurality of actors who engage in policing and controlling the territory through violence and the infliction of fear. Thus, it appears that the old rules of exchange and reliability become blurred and insecure through increasing internal bordering. The relationships between smugglers and their clients, which have been described as a form of “security from below” based on “reciprocity”, solidarity and trust, and social reputation (Sanchez and Zhang, 2018; Zhang et al., 2018) have become more ambiguous, as prices have been rising and new forms of smuggling practices have emerged. Research has shown that anti-immigration measures and enforcement policies have contributed to the changing power relations of the actors in the field of undocumented migration (Stone-Cadena and Álvarez Velasco, 2018; Badillo and Bravo, 2020). This also relates to the desperation of many migrants confronted with new immigration enforcement measures, who are unfamiliar with the routes and conditions and who do not count on reliable social networks and economic resources to engage in more professional smuggling services. Third, the analysis showed how women have become a particular target in the control of undocumented migrants' mobility and their being forced to pay arbitrarily imposed fees in exchange for their bodily integrity. Migrants, including their smugglers, not only have to pay for clandestine border crossings, but throughout the entire transit. Although smugglers are supposed to pass these costs to their clients, migrants must pay higher prices. Women's bodies then become a privileged site of border demarcation through the threat of sexual violence and the symbolic expropriation of their bodies as “spoils of war” by different groups competing for dominance over territory. A violence that serves to discipline the collective of undocumented migrants and families and to manifest the patriarchal power of those groups (Segato, 2014). It is also important to recognize the psychological and social impact of sexual violence on women and their families, given the stigma faced by victims in communities of origin and destination (Girardi, 2008; Ramos, 2017). Finally, border and immigration measures implemented in Mexico have not only contributed to increasing the risks and costs of clandestine travel, but, along with deportations from Mexico, also to processes of impoverishment of families and communities through increasing debt and mistrust between migrants (Nyberg S1ørensen, 2013; Ramos, 2017).

Internal bordering has become an integral part of immigration enforcement throughout Mexico, for example, through the incorporation of civil actors of transport companies into the bordering practices or through implementing deterrence measures such as deportation or protracted administrative procedures through delays in refugee admissions or immigration regularization (Gammeltoft-Hansen and Tan, 2017). Furthermore, these policies contribute to placing people in a hierarchical set of relations (Anthias, 2013, p. 155) which provides space for human rights violations and xenophobia. There is a tendency toward bringing the border into the national territory by retaining migrants in the South, where conditions are particularly dire due to the construction of Central Americans as racialized others (Frank-Vitale and Núñez-Chaim, 2020), and through re-bordering and ‘(social) ordering practices' (Yuval-Davis et al., 2018). Nevertheless, a look at the embodied experiences of migrant women shows that, even though their journeys are marked by precarity and vulnerability, that there is also “resilience and resourcefulness” (Ehrkamp, 2016, p. 2). Scholars have also stressed the importance of understanding the agency in the negotiation of mobility smuggling services (Sanchez and Zhang, 2018; Stone-Cadena and Álvarez Velasco, 2018; Deshingkar, 2019). However, critical migration scholars coincide in stressing that bordering practices translate into everyday violence, which curtails the options of refugees and migrants to choose mobility, access work, or simply confront physical and sexual violence (Yuval-Davis et al., 2018). Violent bordering is productive as it creates ‘disposable' bodies for exploitation and cheap labor (Anthias, 2013; Mezzadra and Neilson, 2013). As Vogt has observed, the commodification of migrant bodies in local economies produces “cargo to smuggle, gendered bodies to sell, labor to exploit, organs to traffic and lives to exchange for cash” (Vogt, 2013, p. 765). Thus, there is a need to open our understanding to the multiple roles that actors can play in the field of power and in this economy of dispossession that draws not only on the need for mobility, but also on violence and fear (Fassin, 2011 in its reflection on coloniality and economy, also Muñoz Martínez et al., 2020).

Since 2018, the United States administration has been looking to incorporate Central American transit countries, such as Guatemala and Honduras, into the securitization and enforcement agendas by implementing safe third-country agreements (Gzesh, 2019). Additionally, during the pandemic, there has been a further push to enforce control measures, such as Title 42 in the United States, the temporary suspension and re-instauration of Migrant Protection Protocols (MPPs) and temporary border closures between countries (Alvarez Velasco, 2021). Moreover, there is a generally high level of discretion in the implementation of different protocols by Mexican and US authorities on undocumented migrants and refugees (Chishti and Bolter, 2020; Ruiz Soto, 2022). If we are to fully understand the dynamics in Mexico, a further look at the policing of migration in Central American countries becomes more relevant to understand the interplay of violence and border enforcement on migrants in transit. Thus, the impact of ongoing securitization on the relationships between different actors in the field, at the local level and in countries of origin, transit, and desired destination remains an important element for further inquiry.

## 7 Conclusions

As the analysis showed, border enforcement at the policy level has an impact on the relationships between people in the field of mobility. Looking at the ways in which migrants' bodily experiences are affected by the policing of these borders is timely, as there is a constant push toward building barriers and walls which prevent people from crossing at various points of migration routes. Violent bordering, or bordering through violence, is productive in many ways, as it weakens the ability of illegalized migrants to negotiate mobility and bodily integrity and fuels local economies which benefit from the commodification of migrants' lives. It also drives an economy of fear and violence which clearly takes advantage of gendered bordering, turning transit territories into territories without rights. Thus, the title citation from our interviewee “They don't care about people; they care about money. How much is a person worth?” speaks of the precarity of transit for people who are left without rights on the sites of internal bordering. It illustrates the fact that the “illegalization” of migrants (including refugees) is a powerful driver for local, national and transnational economies. Yet, these dynamics not only have an impact on individuals, but come with a social cost for societies that are strongly interconnected through transnational ties. In addition, the findings contribute to our understanding of the complexities of border enforcement on the ground and its impact on the social lines of inequality of local and transnational communities, as it changes the ‘rules of the game' (Bourdieu, 1997) and contributes to the commodification of mobility.

## Data availability statement

The datasets presented in this article are not readily available because of privacy and ethical restrictions. Requests to access the datasets should be directed to susanne.willers@gmx.net.

## Ethics statement

Ethical review and approval was not required for the study on human participants in accordance with the local legislation and institutional requirements. The patients/participants provided their written informed consent to participate in this study.

## Author contributions

The author confirms being the sole contributor of this work and has approved it for publication.

## Footnotes

^1^For example “Sin nombre” (Fukunaga, 2009), “Sin Nombre – Life Without Hope” (Mandoki, 2012), “The Golden Dream” (Quemada-Diez, 2013), and documentaries such as “María en tierra de nadie” (Zamora, 2010) by director Marcela Zamora, which depict the living conditions of undocumented Central American migrants in Mexico.

^2^In this article I follow an inclusivist definition of migrants, to refer to all people on the move including refugees (see Carling, 2023).

^3^Beside other political reasons, these groups have become more visible since the year 2017, the end of the US policy called ‘Dry feet, wet feet,' and the end of the policy of issuing “exit trades” to foreigners, which allowed them to leave the country through any border (Torre Cantalapiedra et al., 2021, p. 12).

^4^This is resumed by frequent comments such as “Why are they coming if they know what awaits them?” (“¿*A qué vienen si ya saben lo que les espera?*”), which reverses the victim-victimizer logic, attributing the responsibility for the violence suffered to the victim (for an exhaustive analysis on xenophobic and racist comments in the Mexican media, see Torre Cantalapiedra, 2019).

^5^That measures affected particularly people from Cuba or Haiti and African Countries which before the changes would receive an exit request (“oficio de salida”) from the Immigration Office (INM), after the changes in 2019 this request was changes to a request to leave the country only through the Southern border (Gandini, 2020; Miranda, 2021). A measure that converted these groups in additional clients of smugglers in Mexico.

^6^As “coyotes”, “polleros” or smugglers I refer to people who provide guidance and logistical help to cross borders but also territories in exchange for material compensation in form of money or goods (see also Torre Cantalapiedra and Hernández Campos, 2021, p. 111). Yet, an analysis of women's experiences showed, they are sometimes also expected to pay with sexual “favors” (Ramos, 2017).

^7^As noted by Gandini, and consistent with the Mexican Migration Project, *coyote* employment prices have increased from an average of $600 in the 1990s to $1,000–$1,700 in the early 2000s. This should mainly cover the United States border crossing. Escalating securitization policies in Mexico have contributed to increase costs for crossing Mexico over the last decade. In 2013 my interviewees spoke about having been asked to pay US$ 8,500 for the crossing from Central America. Meanwhile, interviewees from Haiti traversing the Mexican territory spoke of having paid US$9,000 (Gandini, 2020, p. 55, 56). Prices vary according to travel conditions and gender. Elsa Ramos has documented that a groups of three Salvadoran women was asked to pay US$10,000 to travel from El Salvador to the United States (Ramos, 2017).

^8^About 3,000 US$, while the daily Mexican salary was around 5 US$ a day in 2014 (CONASAMI, 2022).

^9^As scholars have shown, in Tapachula, sex workers must pay a 100-peso “tax,” an amount far exceeding the daily minimum wage, which was set at 66 pesos (approximately 4.08US$) in 2015 (CONASAMI, 2022), to the controlling criminal group for every working day (Muñoz Martínez et al., 2020, p. 8).
